# Biological and Physicochemical Characterization of Self-Adhesive Protective Coating Dental Restorative Material after Incorporation of Antibacterial Nanoparticles

**DOI:** 10.3390/polym14204280

**Published:** 2022-10-12

**Authors:** Nazish Gul, Qura Tul Ain Idrees, Muhammad Amber Fareed, Salman Aziz Mian, Hafiz Muhammad Owais Nasim, Fariha Naz, Bashayer Aldahlan, Abdul Samad Khan

**Affiliations:** 1 Department of Science of Dental Materials, Postgraduate Medical Institute, Lahore 54000, Pakistan; 2 Department of Restorative Dentistry, College of Dentistry, Gulf Medical University, Ajman 4184, United Arab Emirates; 3 Department of Dental Materials, Institute of Dentistry, CMH Lahore Medical College, National University of Medical Sciences, Lahore 54000, Pakistan; 4 Department of Restorative Dental Sciences, College of Dentistry, King Khalid University, Abha 62521, Saudi Arabia; 5 College of Dentistry, Imam Abdulrahman Bin Faisal University, Dammam 31441, Saudi Arabia; 6 Department of Restorative Dental Sciences, College of Dentistry, Imam Abdulrahman Bin Faisal University, Dammam 31441, Saudi Arabia

**Keywords:** EQUIA coat, glass ionomer cements, zinc oxide, titanium dioxide, antibacterial, weight measurement, spectroscopic analysis, colony-forming unit, principal component analysis

## Abstract

This study evaluated the physicochemical and antibacterial properties of EQUIA^TM^ coat liquid (E) after incorporation of zinc oxide (ZnO) and titanium dioxide (TiO_2_) nanoparticles. ZnO and TiO_2_ (1 wt.% and 2 wt.%) were dispersed in EQUIA coat. Principal component analysis (PCA) and cluster analysis were performed to visualize systemic variation. Antibacterial activity was evaluated by colony-forming units and crystal violet staining using *Streptococcus* *mutans* and *Lactobacillus* *acidophilus* after 24 h, 48 h, and 72 h, and the microstructure was studied by scanning electron microscopy. The weight change was analyzed at 1 and 21 days. The PCA for TiO_2_- and ZnO-based groups showed 100% variance at all spectral ranges at 600–800/cm and 800–1200/cm, whereas 1200–1800/cm and 2700–3800/cm spectral regions demonstrated 99% variance. The absorbance values were significant (*p* < 0.05) for both nanoparticles-based adhesives, and the specimens with 2 wt.% ZnO showed the maximum response by minimum bacterial attachment, and the control group showed the least response by maximum attachment. The weight change percentage was reduced after the incorporation of antibacterial nanoparticles. It is suggested that EQUIA^TM^ coat containing nanoparticles exhibits promising results, and it may be recommended to clinically use as an improved coating material.

## 1. Introduction

Self-adhesive materials have gained significant attention among dental practitioners [1]. One of the dental adhesive restorative materials is a self-adhesive resin coat that bonds onto the surface of glass ionomer cements (GICs) and provides a perfect seal from extrinsic water to allow complete maturation of GIC that possibly creates a stronger dental restoration [2]. GICs being a water-based material have been widely used in restorative dental sciences. However, despite the development, still this material has certain limitations [3]. These limitations are associated with its low mechanical properties, high moisture uptake, or loss of unbound water at this stage, which highly affects the setting process and results in an opaque restoration that is weak, more soluble, and liable to cracking [4]. The microbial environment, on the other hand, causes morphological changes on GICs, which increase restoration roughness, thus making the restored tooth susceptible to recurrent caries [5]. The mechanical properties have been improved by adding other components such as metal oxides and fibers [6,7]. To address moisture uptake, GIC restoration is coated with bonding resin, petroleum jelly, varnish, or cocoa butter to protect the surface of restoration immediately after its placement [8].

The protective resin coat micromechanically binds and interlocks with the GIC, remaining on the surface for a long time [9]. EQUIA Fil and EQUIA coat from GC, Japan created the EQUIA rapid high-viscosity GIC restorative system. There is evidence that this EQUIA combination with two simple steps, namely GIC bulking filling followed by the application of resin varnish protecting the coating of EQUIA coat, improved clinical longevity, esthetic, physical, biological, and chemical properties of the restoration [10]. EQUIA coat (GC, Tokyo, Japan) is a self-adhesive, wear-resistant, light-cured resin coating material recommended for posterior teeth, highly stress-bearing areas, nonstress-bearing application, and smooth surface restorations [11]. Conventional varnishes provide surface protection for a shorter time, whereas resin coating provides protection for a long period of time, which results in enhanced physical and mechanical performances [12].

Several factors contribute to the short longevity of dental restorations, including the restorative materials’ characteristics, fracture, as well as the oral environment’s complexity, which includes moderate temperature, increased humidity, and nutrients [13]. All of these promote biofilm formation that adheres to tooth surfaces and dental restorations, more favorably to rough restorations [5]. The microcracks open pathways for the cariogenic bacteria resulting in recurrent caries. Since the dental biofilm contains acid-producing bacteria (e.g., *StreptococcuS. mutans* and *Lactobacillus acidophilus),* teeth with restorations are susceptible to recurrent caries mainly at the tooth restoration interface, as the bacterial biofilm damages the adhesive interface [14]. Therefore, the introduction of antibacterial agents into dental restoration is essential to solve the main reason for dental restoration failure (e.g., recurrent caries), which is caused by cariogenic bacteria [15,16].

The antibacterial behavior of zinc oxide (ZnO) and its mechanism of action have been reported when it is present in the form of nanoparticles [17]. ZnO nanoparticles can be partially dissolved into water as Zn ions attribute cytotoxicity to bacteria. ZnO nanoparticles internalized by bacteria can cause cell damage and growth inhibition through electrostatic interactions [18]. Moreover, ZnO has been incorporated into a variety of dental restorative materials to enhance the mechanical and biological properties [19,20]. Increasing the concentration of ZnO nanoparticles in restorative materials results in augmented antimicrobial activity; however, at higher concentrations of the nanoparticles, the depth of light cure of the resin is reduced [21].

Similarly, titanium dioxide (TiO_2_) possesses an antibacterial effect by interfering with phosphorylation, thereby causing oxidative cell death of oral microbes. TiO_2_ nanoparticles have been used as reinforcing agents in dental resin composite restoration because TiO_2_ is a chemically stable inorganic additive, is biocompatible, and has shown improved mechanical and antibacterial properties [22].

Various laboratory-based and clinical trials have been conducted related to EQUIA coat self-adhesive coated material [23,24]; however, studies related to the antibacterial effect of EQUIA coat after incorporating ZnO and TiO_2_ antibacterial nanoparticles are not found. Hence, the research motive was to incorporate two different types of antibacterial nanoparticles to EQUIA coat in order to prepare a new coating to be used for GIC with enhanced antibacterial effects. The main idea of the present study was to assess the chemical, structural, and biological properties of a commercially available GIC coating material EQUIA coat after reinforcement with ZnO and TiO_2_ nanoparticles. The hypothesis of this study was that EQUIA coat™ with nanoparticles would produce the antibacterial effects and eventually lessen the carious activity on the surface of restoration. The null hypothesis was that EQUIA coat™ with nanoparticles lacked the antibacterial efficacy.

## 2. Materials and Methods

EQUIA coat (GC, Tokyo, Japan) Lot no. 1407041 (E) contains methylmethacrylate, colloidal silica, urethane methacrylate, camphorquinone, and phosphate ester monomer. The nanoparticles of ZnO (200–500 nm) and TiO_2_ (40–70 nm) were synthesized through precipitation and sol–gel hydrothermal methods, respectively, as previously described [25,26]. Acetone (Lot no.320110; Sigma Aldrich, St. Louis, MO, USA) was used as a solvent. The nanoparticles of TiO_2_ and ZnO were separately added to EQUIA coat in 1 wt.% and 2 wt.%, respectively. The test microbial strains *StreptococcuS. mutans* (*S. mutans*, ATCC 25175) and *Lactobacillus acidophilus* (*L. acidophilus*, ATCC 4356) were purchased from American Type Culture Collection (ATCC, Manassas, VA, USA), and dehydrated culture media were obtained from Oxoid^TM^ (Basingstoke, Hampshire, UK).

### 2.1. Sample Preparation

EQUIA coat^TM^ GC (E) 1 mL (1.298 g) was dissolved in 0.5 mL acetone at room temperature using a magnetic stirrer (Wigens, Germany) at 100 rpm; then 1 wt.% (0.0129 g) and 2 wt.% (0.0259 g) ZnO and TiO_2_ nanoparticles, respectively, were separately added under continuous stirring at 150 rpm, and acetone was allowed to evaporate for 30 min. Table 1 shows the grouping of the prepared samples.

The experimental group’s disk-shaped (5 mm × 2 mm) specimens were made by pouring the material into a premade brass mold. The disk specimens were covered with a Mylar strip and polymerized for 60 s on both sides with a LED curing unit (Woodpecker, Guilin, China) at 395–480 nm wavelength and an irradiance level of 800 mv/cm^2^. After photo curing was completed, disk-shaped specimens were carefully removed from the molds and smoothly polished on both sides with 800, 1200, 1500, and 4000 grit papers.

### 2.2. Principal Component Analysis and Cluster Analysis

Fourier transform infrared (FTIR) spectra with attenuated total reflectance (ATR) (Thermo Nicolet 6700, Thermo Fisher Scientific, Waltham, MA, USA) were obtained in the region 4000–400/cm by placing the samples (n = 5) on the diamond window of FTIR at a resolution of 8/cm, and an average of 256 scans were collected. Chemometric methods were utilized to assess the spectrum differences of distinct groups included in the data using principal component and cluster analyses. These procedures were carried out by using Unscrambler X 10.2 software (Oslo, Norway). Baseline correction and unit vector normalization were included in the preprocessing. To depict systemic variance in data containing E, EZ1, EZ2, EC-T, and ET2, principal component analysis (PCA) was performed. The method decreases data dimension by projecting it down to a lower dimensional area in directions depicting the greatest variance. A principal component (PC) describes the variance in each direction, and a scores plot depicts the distribution of spectra in relation to the PC. The PCA was carried out over the following spectral ranges: (a) 600–800/cm, (b) 800–1200/cm, (c) 1200–1800/cm, and (d) 2700–3800/cm. Ward’s method was used to perform cluster analysis (CA) over the entire spectral range using squared Euclidean distance.

### 2.3. Antibacterial Study

Two bacterial strains were revived according to the instructions of ATCC. Briefly, after the prescribed incubation time (24 h for *StreptococcuS. mutans* and 48 h for *Lactobacillus acidophilus*), growth culture plates were prepared. For the revival of strains, a sterile Pasteur pipette was used to add a 0.5 mL suitable rehydration medium into the respective ampule. The samples from both tubes were spread on agar plates (brain heart infusion (BHI) broth for *S. mutans* and De Man, Rogosa, and Sharpe (MRS) agar broth for *L. acidophilus*), and these were incubated according to the required conditions (*S. mutans* for 24 h in an aerobic environment in an incubator (Horizontal Flow oven, WOF-155, DAIHAN Scientific Co., Ltd., Seoul, Korea) and *L. acidophilus* at 5–10% CO_2_ in an anaerobic-environment air-tight chamber with anaerobic sachets (Anaerogen Sachets, Thermo Fischer Scientific, Waltham, MA, USA)). For *S. mutans*, BHI broth (Oxoid^TM^ CM 1135 Lot No. 200312) and BHI agar (Oxoid^TM^ CM1136 Lot No. 280329) and, for *L. acidophilus*, MRS broth (Oxoid^TM^ CM 1175 Lot No. 289017) and agar (Oxoid^TM^ CM 0359 Lot No. 444536) media were used. Following the manufacturer’s instructions, 23.5 g agar (BHI) was added into 500 mL of distilled water and stirred at 45 °C. The liquid culture media were synthesized by dissolving 2.22 g BHI broth in 60 mL of distilled water and stirred at room temperature at 100 rpm and autoclaved (Wiseclave, Diahan, Seoul, Korea) at 121 °C for 15 min to prepare the plates; freshly prepared solutions were allowed to cool at room temperature. In order to ensure a standard depth of 4 mm, 20 mL of the prepared solution was poured into each Petri dish and was left to solidify at room temperature for two hours. Culture media for both strains were grown overnight, and after 24 h, the optical density (O.D) was checked with a UV spectrophotometer (Thermo Fischer Scientific, Waltham, MA, USA) and maintained at 0.2 OD for *S. mutans* and 0.1 O.D for *L. acidophilus*.

#### 2.3.1. Colony-Forming Unit (CFU)

To measure the colony-forming units, all the sample disks (n = 5) were immersed in phosphate buffered saline (PBS) pH 7.4 for 1 h at normal room temperature. Each sample was placed in an Eppendorf tube, and 2 mL PBS (BioWORLD Company, Dublin, OH, USA) was added. After 1 h, PBS was removed, and 10 µL of the bacterial culture of *S. mutans* of 0.2 OD was added on each sample and incubated at 37 °C for 1 h. After 1 h, freshly prepared and sterilized 500 µL BHI broth media were added and incubated for 24 h, 48 h, and 72 h. The same procedure was repeated with the *L. acidophilus* strain by maintaining the 0.1 OD and the required incubating conditions of 5% CO_2_ using the MRS media and incubated for 24 h, 48 h, and 72 h. For *S. mutans*, 10^−6^ dilution was used for 24 h plates, and 10^−5^ dilution was selected for 48 h and 72 h plates; 0.1 mL was spread on the BHI agar plate and incubated for 24 h in an incubator at 37 °C. For *L. acidophilus*, 10^−4^ dilution was used for 24 h plates, and 10^−3^ dilution was selected for the 48 h and 72 h incubation time period. Then 0.1 mL was spread on the MRS agar plate and incubated for 48–72 h in an anaerobic environment. After the required time period, the plates were removed from the incubator (WOF-155, Horizontal Flow Oven, DAIHAN Scientific, Seoul, Korea), and bacterial colonies of both strains were visually counted. The number of colonies formed on plates (CFUs) for each tested formulation was converted into CFU/mL by using the formula

CFU/mL = [Colonies number on plates (CFUs)] ÷ [Amount plated × Dilution used](1)

#### 2.3.2. Scanning Electron Microscopy (SEM)

The disk specimens (n = 3) were cultured with their specific strains (*S. mutans* and *L. acidophilus*) by maintaining their environment for a specific timing of 24 h, 48 h, and 72 h. Then the disks were taken out of the media and fixed by dipping in 2.5% formaldehyde for 20 min and then washed with distilled water. After that, the disks were dried in ascending ethanol series 50% EtOH for 5 min, 70% EtOH for 10 min, 96% EtOH for 10 min, and absolute EtOH (100%) for 5 min and then dried at room temperature for two days. All specimens were gold-sputter-coated for 60 s and observed under SEM (TESCAN VEGA-3LMU, Brno, Czech Republic) at 7 kV, and images were taken at different magnifications.

#### 2.3.3. Crystal Violet Staining (CVS)

In crystal violet staining, a 96-well plate was used, and sterilized sample disks (n = 5) were placed in wells in triplicates for each composition and timing. After that, 100 µL of 24 h bacterial culture of 0.2 OD of *S. mutans* and 0.1 OD of *L. acidophilus* was loaded onto disks and incubated for 1 h in an incubator at 37 °C [27]. After 1 h, well plates were removed from the incubator, and 500 µL broth media were added into each well; the plates were incubated again in an incubator for 24 h, 48 h, and 72 h maintaining their required environment. Each disk was washed twice with PBS (pH 7.4). Then 0.5% crystal violet stain was used, and 50 µL of the stain was poured onto each disk and incubated at room temperature for 20 min. Then, the stain was removed from the wells, and the disks were washed with distilled water thrice. Methanol 100% was used and 250 µL was added into each well and incubated for 10–15 min at room temperature. Then 230 µL from each well was shifted to a new well plate, and absorbance was measured at 620 nm using a microplate reader (Elisa, EMR 500, Labomed, Los Angeles, CA, USA).

### 2.4. Weight Measurement Analysis

The method given in ANSI/ADA Specification No. 27-2016 was used to conduct the weight measurement analysis (ISO 4049-2009). A total of 30 (n = 6) samples (5 mm × 2 mm) were weighed and noted as the initial weight *W_i_*. Then all samples were immersed for 1 day and 21 days in 5 mL deionized water at 37 °C. Every third day, the deionized water was replaced. The samples were blotted dry and weighed at both time intervals until equilibrium was reached, and then computed as (*W_f_*). The weight percentage of each specimen was calculated by the formula

Weight measurement % = (*W_f_* − *W_i_* / *W_i_*) × 100 (2)

### 2.5. Statistical Analysis

Data were analyzed using SPSS Statistics for Windows Software, version 21 (IBM Software, Armonk, NY, USA). Descriptive statistics were used to calculate the maximum, minimum, mean, and standard deviation values. The normality of the data was assessed by the Shapiro–Wilk test. Comparison of two and more than two groups was performed by the one-way analysis of variance (ANOVA) test followed by post hoc Tukey’s test. Repeated measurement analysis was also carried out for weight measurement analysis. All the *p* values of less than 0.05 were considered statically significant.

## 3. Results

### 3.1. Principal Component Analysis and Cluster Analysis

**Figure 1:**
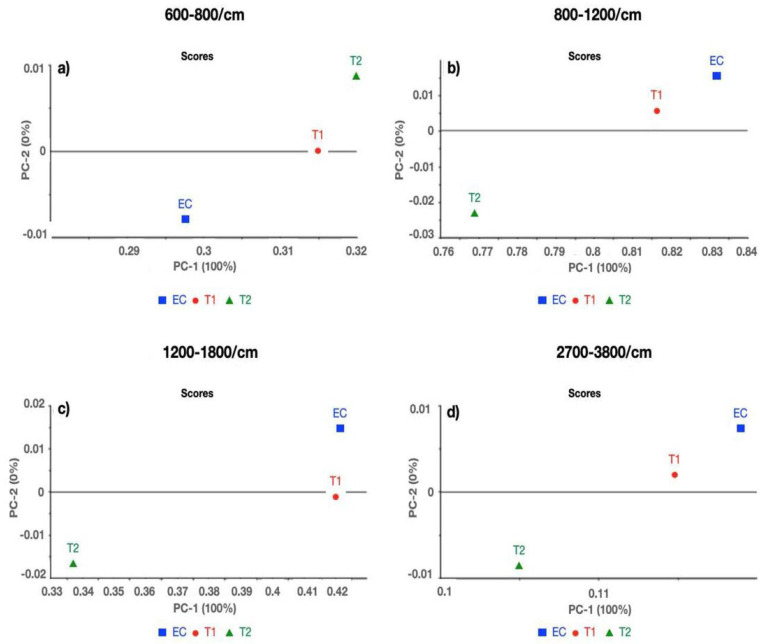
Principal component analysis (PCA) of EQUIA coat (E), 1% TiO_2_ (ET1), and 2% TiO_2_ (ET2) at spectral ranges (**a**) 600–800/cm, (**b**) 800–1200/cm, (**c**) 1200–1800/cm, and (**d**) 2700–3800/cm.

**Figure 2:**
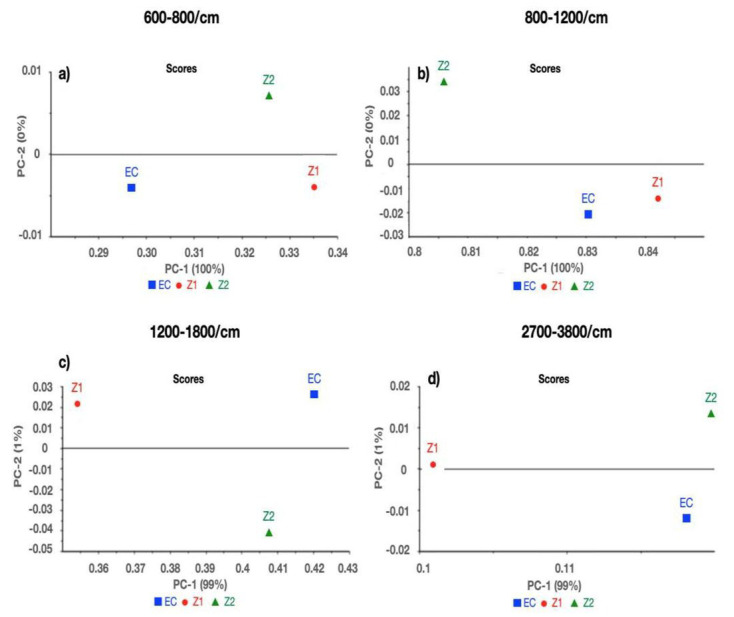
Principal component analysis (PCA) of EQUIA coat (E), 1% ZnO (EZ1), and 2% ZnO (EZ2) at spectral ranges (**a**) 600–800/cm, (**b**) 800–1200/cm, (**c**) 1200–1800/cm, and (**d**) 2700–3800/cm.

Two comparisons were conducted between (a) E, ET1, and ET2; and (b) E, EZ1, and EZ2 at the following spectral ranges: (a) 600–800/cm, (b) 800–1200/cm, (c) 1200–1800/cm, and d) 2700–3800/cm. Excellent separation between E, ET1, and ET2 at spectral ranges (a) 600–800/cm, (b) 800–1200/cm, (c) 1200–1800/cm, and (d) 2700–3800/cm was observed with 100% variance in the first component (Figure 1a–d). While comparing E, EZ1, and EZ2, 100% variance was observed at 600–800/cm and 800–1200/cm, whereas 1200–1800/cm and 2700–3800/cm spectral regions demonstrated 99% variance in the first component (Figure 2a–d).

**Figure 3:**
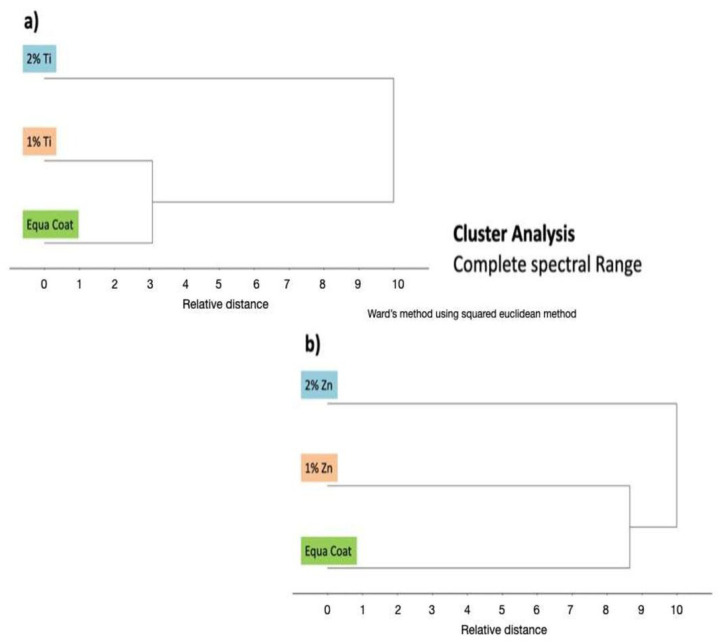
Cluster analysis (CA) at complete spectral range. (**a**) EQUIA coat (E), 1% TiO_2_ (ET1), and 2% TiO_2_ (ET2) (**b**) EQUIA coat (E), 1% ZnO (EZ1), and 2% ZnO (EZ2).

**Figure 4:**
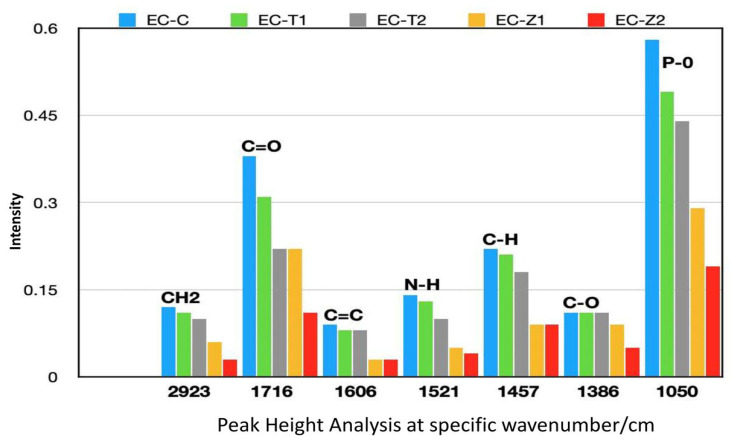
Peak height analysis of control (E) and experimental samples (EZ1, EZ2, ET1, and ET2)) at various regions.

Sample groupings and inner group variations were observed using cluster analysis (Figure 3a,b), which complemented the findings of the principal component analysis. Maximum differences were observed in E and EZ1 at the complete spectral range (Figure 3b). However, exceptional separation was overall recorded between the comparisons (a),(b). The FTIR spectra analyses of the peak height of the control and experimental samples are shown in Figure 4. It was found that the intensities of peak groups (C–H, C=O, C=C, N–H, and C–O–C) were reduced after incorporating ZnO and TiO_2_ in EC.

### 3.2. Antibacterial Analysis

#### 3.2.1. Colony-Forming Unit (CFU)

The comparative CFUs of *L. acidophilus* and *S. mutans* at 24 h, 48 h, and 72 h are given in Table 2, and the one-way ANOVA test that was used to determine the mean difference in the number of colonies among groups showed that the difference was significant in both groups, i.e., *L. acidophilus* and *S. mutans* with *p*-value < 0.001. 

##### CFU at 24 h

For multiple comparisons, post hoc Tukey’s test showed that the difference among all experimental groups at 24 h was statistically significant (*p* ˂ 0.05). However, the difference between EZ2 and ET1 was statistically not significant (*p* ˃ 0.05). The mean number of colonies of *L. acidophilus* in E was lower compared with those in the remaining groups. In *S. mutans*, the mean numbers of colonies of E and EZ1 were lower compared with those of the remaining groups, and between these two groups, E showed the minimum CFU, whereas ET2 showed the highest mean values for the number of CFUs. Comparing all the experimental and control groups, it was showed that a significant difference exists (*p* ˂ 0.05), and no significant difference was found between E and EZ1 (*p* ˃ 0.05). In *S. mutans*, the mean number of colonies of E was lower compared with those of the remaining groups; however, both experimental and control groups in pairwise combination showed a significant difference (*p* ˂ 0.05).

##### CFU at 48 h

At 48 h, the mean number of colonies of all groups was significant (*p*-value< 0.001) in both organisms, i.e., *L. acidophilus* and *S. mutans*. For multiple comparisons, post hoc Tukey’s test presented that all comparisons showed a significant difference (*p* ˂ 0.05) except EZ1, and E, EZ2, and ET1 showed a nonsignificant difference (*p* ˃ 0.05). The mean numbers of colonies at E and the experimental group EZ1 were lower compared with those of the remaining groups in the *L. acidophilus* organism, whereas ET2 showed the highest mean values for the number of colonies. In *S. mutans*, the mean number of colonies at E was lower compared with those of the remaining groups as shown in Table 2, and both experimental and control groups in pairwise combination showed a significant difference (*p* ˂ 0.05).

##### CFU at 72 h

At 72 h, the difference among groups was significant in both organisms with *p*-value < 0.001. For multiple comparisons, post hoc Tukey’s test showed that, with *L. acidophilus*, the mean number of colonies of all comparisons showed significant difference (*p* ˂ 0.05), except for EZ1 and ET1, EZ1 and E, and ET1 and E, which were statistically nonsignificant (*p* ˃ 0.05). In *S. mutans*, all relations showed a significant difference (*p* ˂ 0.05), and a nonsignificant difference was found only between EZ1 and E, as well as between EZ2 and ET1 (*p* ˃ 0.05). In both organisms, the mean number of colonies in the E group was minimum, and that in ET2 was maximum.

#### 3.2.2. Scanning Electron Microscopy (SEM)

**Figure 5:**
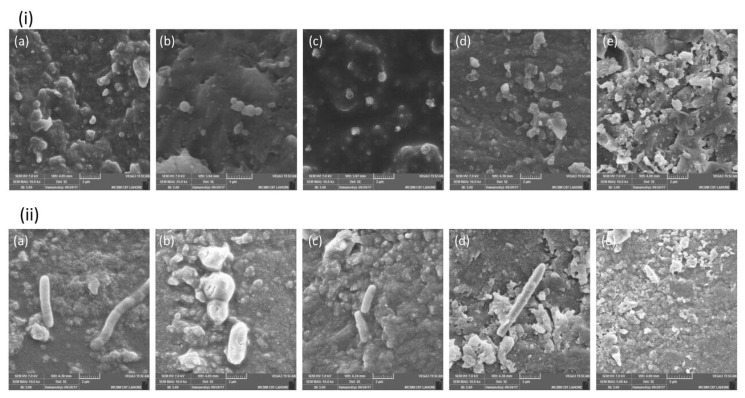
SEM images showing attachment of (**i**) Streptococcus *mutans* and Lactobacillus *acidophilus* (**ii**) on (**a**) E (control), (**b**) ET1, (**c**) ET2, (**d**) EZ1, and (**e**) EZ2 at 48 h. Representative images were taken at 7 kV, and magnification was 10k×.

The representative comparative SEM images of the experimental groups (EZ1, EZ2, ET1, and ET2) and the control group (E) at 48 h for viewing the morphology and attachment of *L. acidophilus* and *S. mutans* are shown in Figure 5. *S. mutans* appeared in the form of individual cocci or in aggregates on both experimental and control disks with prominent presence in abundance on E samples in the form of piles. On the other hand, *L. acidophilus* appeared in the form of rods of bacilli mostly scattered on the disk. Few areas were also observed on the disks where no bacteria were present. From the SEM images, it was observed that more bacteria were attached with the control group (E) sample disks as compared with the experimental disks (EZ1, EZ2, ET1, and ET2). In comparison with EZ1 and EZ2, more bacterial adherence was observed with ET1 and ET2, whereas EZ1 was more prone to bacterial attachment as compared with the sample disks of EZ2. Similarly, there was more attachment on the sample disks of ET1 as compared with ET2. 

#### 3.2.3. Crystal Violet Staining (CVS)

A comparison of the absorbance values of *L. acidophilus* and *S. mutans* at 24 h, 48 h, and 72 h is given in Table 3. The statistical difference at 24 h among groups was significant in both organisms with *p*-value < 0.001. For multiple comparisons, post hoc Tukey’s test showed that the mean absorbance values of the relations EZ1 and E, EZ1 and ET1, EZ2 and ET1, EZ2 and E, and ET1 and ET2 were statistically significant (*p* ˂ 0.05), whereas other relations showed a nonsignificant difference (*p* ˃ 0.05) in the *L. acidophilus* organism. In *S. mutans*, all the relations showed a significant difference (*p* ˂ 0.05). In both bacterial strains, the E group showed the highest absorbance values, and EZ2 showed the lowest values.

At 48 h, a comparison of the absorbance values of *L. acidophilus* and *S. mutans* showed that the difference among groups was significant in both organisms with *p*-value < 0.002 and <0.001, respectively; for multiple comparisons, post hoc Tukey’s test showed that the mean absorbance values of the relations EZ1 and EZ2, EZ2 and ET1, and ET1 and ET2 showed a significant difference (*p* ˂ 0.05), whereas the other relations were nonsignificant (*p* ˃ 0.05) in *L. acidophilus*. In *S. mutans*, the mean absorbance values of EZ1 and EZ2, EZ1 and E, EZ2 and ET1, EZ2 and E, ET1 and E, and ET2 and E showed a significant difference (*p* ˂ 0.05), whereas other relations were nonsignificant (*p* ˃ 0.05).

At 72 h, a comparison of the absorbance values of *L. acidophilus* and *S. mutans* showed that the difference among groups was significant in both organisms with *p*-value < 0.002 and <0.001, respectively. For multiple comparisons, post hoc Tukey’s test showed that the mean absorbance values of the relations EZ1 and EZ2, EZ2 and ET1, EZ2 and E, and ET2 and E were significant (*p* ˂ 0.05) in *L. acidophilus*. In *S. mutans*, the absorbance values of all the relations showed a significant difference (*p* ˂ 0.05), whereas the comparison of EZ2 and ET2 and EZ1 and ET1 was nonsignificant (*p* ˃ 0.05) as shown in Table 3.

### 3.3. Weight Measurement Analysis

**Figure 6:**
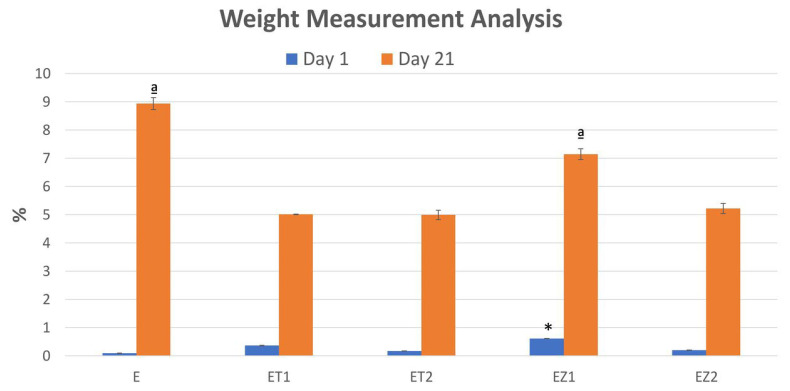
Graph showing mean measurement of weight change % after 1 and 21 days. * Statistically significant difference at day 1; ª significant difference than other groups.

The measurement of weight change after 1 day and 21 days is given in Figure 6. It was found that at day 1, EZ1 showed a significantly higher weight-change percentage as compared with the other groups. On the other hand, no significant difference was observed in the remaining groups. At day 21, the statistical difference among groups was significant with *p*-value = 0.001. The mean weight-change percentage of group E was significantly higher as compared with those of the ET1, ET2, and EZ2 groups. Meanwhile, no significant difference was observed between groups E and EZ1. The results of the repeated-measures ANOVA test revealed that the weight-change percentage was significantly increased from day 1 to day 21 in each group.

## 4. Discussion

The structural, physical, and antibacterial properties of a self-adhesive polymer (EQUIA coat) with and without reinforcement of TiO_2_ and ZnO nanoparticles in two distinct weight percentages were compared in this work. An antimicrobial action of coating materials is desired to prevent cariogenic bacterial colonization. This result can be accomplished by including antibacterial agents into the formulation of the adhesive system [28]. Zinc oxide and titanium dioxide having antibacterial properties are effective in countering numerous types of dental plaque bacteria including *L. acidophilus* and *S. mutans*. The oral biofilm contains variable and complex microbial communities; among these, *S. mutans* and *L. acidophilus* have been implicated as the major pathogens in dental caries [14]. Therefore, *S. mutans* and *L. acidophilus* were investigated in the present study. *S. mutans* is known as the main ethological bacterium of dental caries initiation, and it plays a significant role in the colony formation of an existing biofilm; it is also specifically considered the predominant microorganism in enamel caries [29]. On the other hand, the essential bacterium of caries progression and secondary caries formation is *L. acidophilus* [30].

The addition of zinc oxide and titanium dioxide nanoparticles in dental restorative materials improves not only antibacterial qualities but also physical and mechanical properties such as microhardness, compressive strength, and flexural strength [19,31]. In the present study, zinc oxide and titanium dioxide nanoparticles were separately incorporated in different concentrations, whereby the hypothesis is accepted that antibacterial performance was improved after adding these nanoparticles.

The results of the structural analysis showed that there was a remarkable reduction in the peak heights of carbonyl C=O (EZ1~0.22, EZ2~0.11), aromatic C=C (EZ1~0.03, EZ2~ 0.03), and amide N–H (EZ1~0.05, EZ2 ~0.04) in EZ1 and EZ2 groups as compared with ET1 and ET2, respectively. The change in the peak heights of different chemical groups in these experimental groups is shown as follows: carbonyl C=O (ET1~ 0.31, ET2~0.22), aromatic C=C (ET1~0.08, EZ2~0.08), and amide N–H (ET1~0.13, ET2~0.10). The shift in the peak heights could be due to either the accumulation of these functional groups or the interaction of the functional groups with nanoparticles (ZnO and TiO_2_). Multivariate data analysis (PCA and CA) complemented the findings of FTIR spectroscopy. While differentiating the control group from TiO_2_ and ZnO, PCA proved to be extremely sensitive in separating different samples with a maximum of 100% and a minimum of 99% variance. PCA results, based on spectroscopic assignments, have demonstrated differences between control and various concentrations of TiO_2_ and ZnO. Similarly, these groups have been distinguished over the complete spectral range using cluster analysis.

The development of the oral biofilm on the surface of restoration is the main reason for the secondary caries. Thus, it is necessary to rule out the degree of bacterial adhesion on the surface of dental biomaterials [32]. EQUIA coat is a new coating material, and available research data are very limited to analyze its properties. Furthermore, it does not give information about its antibacterial potential, on this account. Therefore, we evaluated the antibacterial properties of EQUIA coat with zinc oxide and titanium dioxide nanoparticles against *S. mutans* and *L. acidophilus*, as both have a significant role in caries inhibition [33]. Plaque, being the major reason for caries, is a locally acting causative agent for carious activity; therefore, the use of local antimicrobial agents is more effective as compared with systemic ones [34].

Zinc oxide nanoparticles have a broad-spectrum bactericidal activity among nanomaterials; however, their antimicrobial activity is largely dependent upon their size and concentration. Their unique properties of low toxicity to humans, white color, UV-light blocking, and ability to prevent biofilm formation make them suitable to be used as coating materials for biomedical and other fields [35]. Titanium dioxide is an antibacterial metal oxide that is widely known for its efficacy against both Gram-positive and -negative organisms. However, because titanium dioxide is photocatalytic, its toxicity is primarily reliant on its exposure to UV radiation [36].

In this study, it was proposed that the incorporation of zinc oxide and titanium dioxide nanoparticles at concentrations of 1 wt.% and 2 wt.% into EQUIA coat would increase its antibacterial properties against *S. mutans* and *L. acidophilus*. These two concentrations were selected in accordance with the results of previous studies [37,38], which suggested that, by increasing the nanoparticle concentrations, the antibacterial properties of the materials increase; however, the depth of cure decreases, which consequently decreases the physical and mechanical properties of restorative materials. In addition to that, similar findings were detected in the present study where we initially used higher concentrations of these nanoparticles in the pilot studies. High concentrations (i.e., 3 wt.%, 5 wt.%, and 10 wt.%) of both zinc oxide and titanium dioxide showed hindrance in curing of photoactivated EQUIA coat. Therefore, 1 wt.% and 2 wt.% of nanoparticles were selected to study the physicochemical and biological properties.

The results of this present study showed that the concentration of EZ and ET (1 wt.% and 2 wt.%) did not give much antibacterial properties. From the data collected, it has been cleared that the colonies formed by EZ1 are comparable with those by the control group; however, the colonies were less in EZ2, whereas ET2 showed the maximum colonies on the plate independent of time. These results are in contrast with other results obtained from previous studies in which zinc oxide and titanium dioxide nanoparticles behaved as antimicrobial agents [21,38].

The extracellular matrix is released when zinc oxide and titanium dioxide nanoparticles damage the bacterial cell membrane, causing the bacteria to die [39,40]. Zinc oxide and titanium dioxide nanoparticles are insoluble and can be used as fillers with or without nanosilica particles in order to reinforce the composite materials. There is another concept in which the production of reactive oxygen species by titania and some other metal oxides in the presence of ultraviolet light accounts for their special properties, and they are unable to inhibit bacterial growth in the absence of light. This photocatalytic behavior can be attributed to both nanoparticles as both produce antimicrobial oxygen species under ultraviolet light [21].

There exists a direct relationship between the concentration of nanoparticles and antimicrobial activity of restorative materials, which leads to the fact that concentrations of nanoparticles (1 wt.% and 2 wt.%) used in this study were not enough to give antibacterial effects. According to the results of many studies, GICs with EQUIA coating showed increased bacterial adhesion as compared with other restorative materials [41,42]. The results of the present study negate the antibacterial activity of the control group EQUIA coat. The observations presented in this study and the work by Garcia et al. showed that the addition of nanoparticles may not increase the antibacterial activity of restorative materials [43].

Crystal violet staining assay, which is based on the absorbance measurement, is the most commonly used technique for determining biofilm formation in in vitro studies. The quantification of biofilm formation by measuring OD and counting the viable bacteria on agar plates as well as the absorbance values by a CV staining protocol provided the reproducible results and allowed the evaluation of bacterial growth on the specimen surface. The results of CV staining showed significantly higher absorbance values for EQUIA coat as compared with experimental groups containing nanoparticles, except the experimental group containing TiO_2_ 1 wt.%, which showed close resemblance of absorbance values to those of the control group; on the other hand, EQUIA coat with zinc oxide 2 wt.% appeared to be the most appropriate concentration with minimum bacterial adhesion. The nanoparticles of zinc were more active with less bacterial adhesion as compared with titanium dioxide nanoparticles, and these results are similar with the results of Besinis et al.’s study, whereby titanium dioxide exhibited more bacterial adherence and growth as compared with other nanoparticles [44].

In comparison with nanoparticle compositions, scanning electron microscopy revealed that attached bacteria were more prevalent on resin coatings. For *S. mutans* and *L. acidophilus*, increased bacterial adhesion was observed with time, and maximum adhesion was found at 72 h. The shape and range of the measured dimensions obtained for the isolated bacteria were in good agreement with the results of other researchers [45]. Initially, an increase was observed in the bacterial growth after 24 h followed by reduction in the bacterial count at 48 h, and this decrease was almost stable at 72 h with little variation in the number of colonies for both strains. Numerous factors have been identified that influence the formation of the biofilm, and surface roughness is considered the most important factor as it favors the bacterial adhesion and resultant formation of biofilm mass on the surface of restoration [46]. In the present study, it was attempted to remove the roughness on the sample surface by using grit paper. Additional physicochemical characteristics such as crystallinity, surface hydrophobicity, and surface energy are other factors that may also influence the bacterial adhesion [47].

Naguib et al. reported the antibacterial activity of ZnO- nanoparticles against *Staphylococcus aureus*, and no bacterial growth was observed after 7 days [48]. The results of this present study are similar to those of Naguib et al.; however, we conducted antibacterial activity analysis for 72 h only. Therefore, it is recommended to conduct studies for a longer duration for better understanding of antibacterial behavior at a longer time. The results of the present study show better antibacterial behavior of ZnO as compared with TiO_2_, and significant reduction of the CFU of *S. mutans* was observed in comparison with *L. acidophilus*.

The EQUIA coat material investigated in the present study consisted of UDMA and PMMA. After uptake of water, highly hydrophilic monomers such as methacrylate may exceed the polymerization shrinkage value. This could be due to the high values of the stress reduction due to hygroscopic relaxation caused by water sorption [49,50]. Metallic nanoparticles added to composites also improve the material’s stability [51]. Therefore, the weight change of experimental groups was lower than that of the control group, and synthesized groups were stable than the control group. Components of a resin-based material (short-chain polymers and residual monomers, metallic ions) can elute into oral salivary fluids, where they can interact with mucosal tissue or diffuse to dentin and pulpal tissue [52].

This study was the first attempt to analyze EQUIA coat^TM^ on antibacterial grounds. The results obtained regarding bacterial adhesion to this coating material are acceptable, as it will lead to less carious lesion. However, more studies are needed to confirm its antibacterial potential, and researchers should be encouraged to work on this material and to explore more of its properties. The limitations of the study can be the manual mixing of materials, size limitations of nanoparticles, and duration of the study. Moreover, the variables in the oral cavity cannot be completely reproduced in an in vitro setting. Absence of natural saliva in the laboratory setting and fluid within dentin also serve as limiting factors. Another consideration is that bacteria present in the oral biofilm tend to be less susceptible as compared with planktonic bacteria. Therefore, further testing and in vivo trials are required for the extrapolation of present findings in the clinical environment.

## 5. Conclusions

EQUIA coat was reinforced with ZnO and titanium dioxide by using physical mixing with in situ polymerization process. The infrared spectroscopic and scanning electron microscopy analysis confirmed that the increase in nanoparticle concentration results in the spectral peaks becoming less intense, and reduction in the heights of methacrylate, carbonyl, urethane, and phosphate groups occurred. The addition of antibacterial nanoparticles to EQUIA coat affects the bacterial adhesion as compared with the control group. Among zinc oxide and titanium dioxide nanoparticles, zinc particles showed more decrease in bacterial attachment and biofilm formation, whereby EZ2 proved to be the most appropriate composition to achieve the decreased bacterial adherence as compared with EZ1, ET1, and ET2. With increasing time period from 24 h to 72 h, bacterial attachment increased on all composition disks with more prominent abundance on control samples. Colony-forming-unit results showed more colonies on the plates of experimental compositions in comparison with the control groups (E). ET2 exhibited the increased bacterial growth at 24 h, 48 h, and 72 h, whereas EZ1 showed the decreased growth of colonies.

## Figures and Tables

**Table 1:** Composition and abbreviations of control and experimental groups used.

Sr. #	Experimental Groups	Wt.% of Nanoparticles
1	E	EQUIA coat—Control
2	ET1	TiO_2_ 1 wt.% + E
3	ET2	TiO_2_ 2 wt.% + E
4	EZ1	ZnO 1 wt.% + E
5	EZ2	ZnO 2 wt.% + E

**Table 2:** Mean number of colonies (CFUs) of *Lactobacillus acidophilus* and *StreptococcuS. mutans* at 620 nm by ELISA at 24 h, 48 h, and 72 h.

Organism	Time	Groups	Mean ± SD	*p*-Value
*Lactobacillus* *acidophilus*	24 h	EZ1	1.15 × 10^7^ ± 0.06 × 10^7^	<0.001 *
		EZ2	1.62 × 10^7^ ± 0.05 × 10^7^	
		ET1	1.49 × 10^7^ ± 0.07 × 10^7^	
		ET2	1.84 × 10^7^ ± 0.07 × 10^7^	
		E	0.92 × 10^7^ ± 0.08 × 10^7^	
*StreptococcuS. mutans*	24 h	EZ1	6.53 × 10^8^ ± 0.56 × 10^8^	<0.001 *
		EZ2	12.1 × 10^8^ ± 0.61 × 10^8^	
		ET1	9.37 × 10^8^ ± 0.61 × 10^8^	
		ET2	17.2 × 10^8^ ± 0.65 × 10^8^	
		E	5.23 × 10^8^ ± 0.90 × 10^7^	
*Lactobacillus* *acidophilus*	48 h	EZ1	1.50 × 10^6^ ± 0.06 × 10^6^	<0.001 *
		EZ2	1.83 ×10^6^ ± 0.07 × 10^6^	
		ET1	1.97 × 10^6^ ± 0.05 × 10^6^	
		ET2	2.52 × 10^6^ ± 0.06 × 10^6^	
		E	1.35 × 10^6^ ± 0.06 × 10^6^	
*StreptococcuS. mutans*	48 h	EZ1	1.77 × 10^8^ ± 0.05 × 10^8^	<0.001 *
		EZ2	2.51 × 10^8^ ± 0.07 × 10^8^	
		ET1	2.03 × 10^8^ ± 0.08 × 10^8^	
		ET2	2.88 × 10^8^ ± 0.12 × 10^8^	
		E	1.09 × 10^8^ ± 0.07 × 10^8^	
*Lactobacillus* *acidophilus*	72 h	EZ1	1.24 × 10^6^ ± 0.05 × 10^6^	<0.001 *
		EZ2	1.59 × 10^6^ ± 0.07 × 10^6^	
		ET1	1.32 × 10^6^ ± 0.06 × 10^6^	
		ET2	2.19 × 10^6^ ± 0.07 × 10^6^	
		E	1.21 × 10^6^ ± 0.06 × 10^6^	
*StreptococcuS. mutans*	72 h	EZ1	1.20 × 10^8^ ± 0.05 × 10^8^	<0.001 *
		EZ2	2.21 × 10^8^ ±0.05 × 10^8^	
		ET1	2.17 × 10^8^ ± 0.04 × 10^8^	
		ET2	2.55 × 10^8^ ± 0.05 × 10^8^	
		EC	1.29 × 10^8^ ± 0.07 × 10^8^	

* significant difference.

**Table 3:** Absorbance values of Lactobacillus *acidophilus* and Streptococcus *mutans* at 620 nm by ELISA at 24 h, 48 h, and 72 h.

Organism	Time	Group	Mean ± SD	*p*-Value
*Lactobacillus* *acidophilus*	24 h	EZ1	0.183 ± 0.011	<0.001 *
		EZ2	0.157 ± 0.008	
		ET1	0.241 ± 0.017	
		ET2	0.196 ± 0.012	
		E	0.236 ± 0.026	
*StreptococcuS. mutans*		EZ1	0.524 ± 0.013	<0.001 *
		EZ2	0.382 ± 0.018	
		ET1	0.595 ± 0.009	
		ET2	0.460 ± 0.029	
		EC	0.696 ± 0.020	
*Lactobacillus acidophilus*	48 h	EZ1	0.536 ± 0.008	0.002 *
		EZ2	0.371 ± 0.058	
		ET1	0.571 ± 0.009	
		ET2	0.446 ± 0.058	
		E	0.478 ± 0.050	
*StreptococcuS. mutans*		EZ1	0.632 ± 0.022	<0.001 *
		EZ2	0.568 ± 0.027	
		ET1	0.641 ± 0.027	
		ET2	0.585 ± 0.008	
		E	0.832 ± 0.017	
*Lactobacillus acidophilus*	72 h	EZ1	0.735 ± 0.036	0.002 *
		EZ2	0.613 ± 0.021	
		ET1	0.739 ± 0.023	
		ET2	0.626 ± 0.078	
		E	0.756 ± 0.027	
*StreptococcuS. mutans*		EZ1	0.738 ± 0.016	<0.001 *
		EZ2	0.607 ± 0.023	
		ET1	0.715 ± 0.023	
		ET2	0.648 ± 0.023	
		E	0.938 ± 0.016	

* significant difference.
